# Surgical Treatment Methods of Urolithiasis in the Pediatric Population

**DOI:** 10.34763/devperiodmed.20182201.8893

**Published:** 2018-04-12

**Authors:** Joanna Samotyjek, Beata Jurkiewicz, Andrzej Krupa

**Affiliations:** 1 Pediatric Surgery and Urology Clinic CMKP in Dziekanów Leśny, Leśny Poland

**Keywords:** urolithiasis, children, ESWL, URSL, PCNL, RIRS, kamica układu moczowego, dzieci

## Abstract

Urolithiasis in the pediatric population represents a major challenge associated with both the diagnosis and therapy of the condition. Over the past 25 years, the incidence has increased. The average age of pediatric patients with stones is about 7-8 years and the recurrence rate is 24%-50%. More than 80% of the stones are eliminated spontaneously. The remaining ones require conservative or surgical treatment. Choosing the most appropriate treatment depends on many factors. Surgical procedures in children are the same as in adults. These include extracorporeal shockwave lithotripsy (ESWL), ureterolithotripsy (URSL), retrograde intrarenal surgery (RIRS), percutaneous nephrolithotomy (PCNL) and laparoscopic or open surgery. ESWL is a method of choice for the treatment of stones with a diameter of ≤20 mm located in the upper urinary tract, while PCNL is used in the treatment of deposits ≥1.5 cm located in the upper pole of the kidney, deposits of ≥1.0 cm located in the lower pole of the kidney, as well as hard stones such as cystic or struvite ones. URSL/RIRS is a method for ureteral and renal stones. Open surgery is indicated in cases when anatomical anomalies coexist with urolithiasis, or when the use of PCNL or ESWL is impossible. The ideal procedure should be effective, safe and allow the complete evacuation of the stones after the 1st procedure.

## Streszczenie

Kamica układu moczowego u dzieci stanowi duże wyzwanie zarówno diagnostyczne jak i terapeutyczne. W ciągu ostatnich 25 lat zachorowalność wzrosła. Średni wiek pacjenta pediatrycznego z kamicą układu moczowego to 7-8 lat. Ponad 80% złogów wydalanych jest spontanicznie. Pozostałe wymagają leczenia zachowawczego lub chirurgicznego. Wybór najbardziej odpowiedniej metody leczenia zależy od wielu czynników. Procedury chirurgiczne u dzieci są takie same jak u dorosłych, tj. litotrypsja pozaustrojowa (ESWL), ureterolitotrypsja (URSL), ureterorenofiberoskopia (RIRS), przezskórna nefrolitotrypsja (PCNL), chirurgia otwarta lub laparoskopowa. ESWL jest metodą z wyboru w leczeniu złogów mniejszych niż 20mm i zlokalizowanych w górnym biegunie nerki. PCNL jest metodą z wyboru w leczeniu kamieni ≤1,5 cm zlokalizowanych w górnym biegunie nerki, kamieni ≤1 cm zlokalizowanych w dolnym biegunie nerki lub kamieni twardych np. cystynowych lub struwitowych. URSL jest metodą stosowaną w usuwaniu złogów z moczowodu. Chirurgia otwarta jest stosowana w przypadkach, gdy anomalie anatomiczne współistnieją z kamicą lub gdy wykonanie ESWL lub PCNL jest niemożliwe. Optymalna procedura powinna umożliwić efektywne, bezpieczne i całkowite ewakuowanie złogów.

## Introduction

Urolithiasis in the pediatric population represents a major challenge associated with both the diagnosis and therapy of the condition. Over the past 25 years, the incidence has increased [1, 2, 3, 4, 5, 6, 7]. Higher morbidity is due to poor dietary habits (diet rich in sodium, rich in protein, insuffcient supply of fluids), obesity, hypertension, environmental pollution, acceleration of life, uncontrolled supply of multivitamins and dietary supplements [8, 9, 10]. At the same time improving the quality of diagnostics, the increasing frequency of ultrasonography and CT studies at admissions offces during the diagnosis of abdominal and lumbar pain significantly contributed to the increase in the detection of stones [11]. Kidney disease refers to patients of all ages. In the literature there are reports of 4-day neonates with diagnosed kidney stones, but the average age of pediatric patients with stones is about 7-8 years [11]. It is a recurrent condition. In retrospective studies, recurrences occur in 24% to 50% of the patients [12]. The highest rate of recurrence is observed in children with metabolic disorders. It is therefore essential to choose a treatment method in children that would allow the least invasive and most e&ective removal of stones. More than 80% of all stones are eliminated spontaneously and do not require any intervention [13]. The remaining ones require conservative or surgical treatment. Choosing the most appropriate treatment depends on many factors, such as the location, size and composition of the stones, the age of the patient, anatomical conditions, urinary flow problems, or recurrent urinary tract infections [14]. The presence of stones in the urinary tract does not determine the necessity of surgical intervention. According to Van Savage, stones smaller than 4 mm not causing retention should be observed and treated only conservatively. It was noticed that also stone composition has a relevant influence on spontaneous evacuation. Calcium phosphate stones were spontaneously evacuated in 78% of all patients, whereas 91% of calcium oxalate stones required surgical treatment. Cystine and struvite stones usually require surgical treatment because of their stability and large sizes [15]

Surgical procedures for the treatment of stone disease in children are the same as in adults. These include ESWL, URSL, RIRS, PCNL and laparoscopic or open surgery.

## Extracorporeal shockwave lithotripsy (ESWL)

**Fig. 1:**
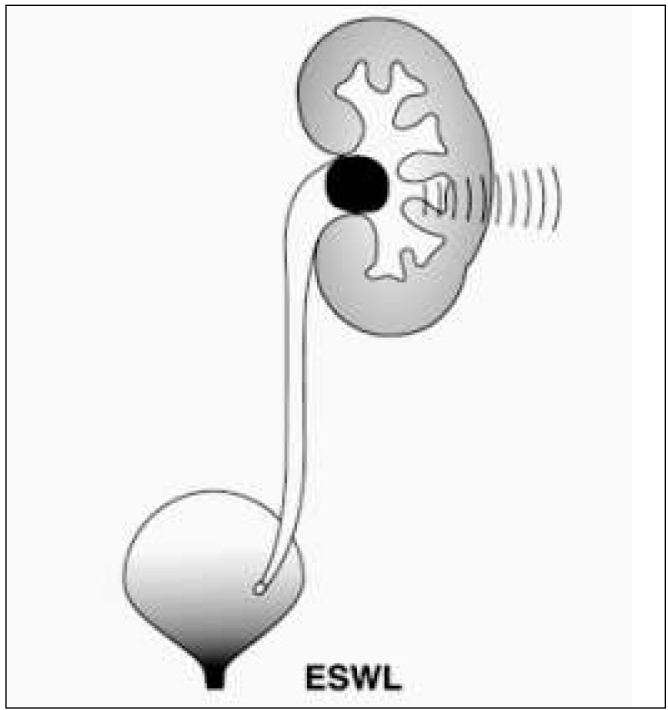
ESWL procedure diagram. Ryc. 1. Schemat procedury ESWL.

**Fig. 2:**
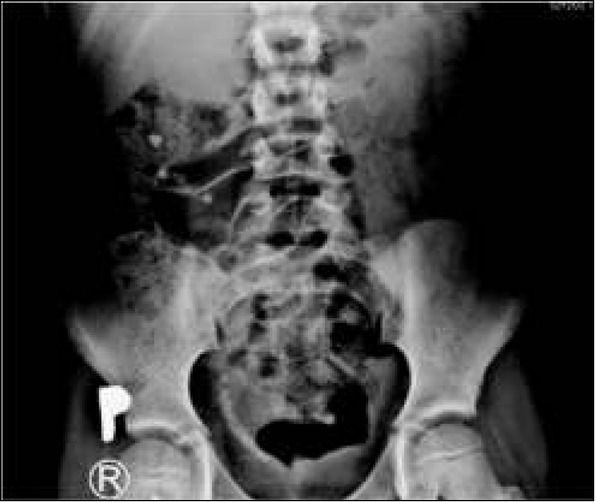
Plain X ray – “stein strasse”. Ryc. 2. Przeglądowe RTG jamy brzusznej – „stein strasse”.

ESWL stands for extracorporeal shockwave lithotripsy (Figure 1). During this procedure stones are divided into smaller fragments under the impact of the shock wave which is focused on them. There are di&erent kinds of lithotripters, i.e.: electrohydraulic, piezoelectric and electromagnetic ones, depending on the mechanism of shockwave generation. The shockwave is focused on the stone under X-ray, or in the pediatric population preferably under ultrasound control. Patients under 10 years old require anesthesia to avoid uncontrolled patient movement. As recommended by the EAU, in children ESWL is a method of choice for the treatment of stones with a diameter of ≤20 mm located in the upper urinary tract. The e&ectiveness of the method varies from 68% to 92% depending on the center [16]. It depends on many variables. Stones longer than 15 mm, in long renal tubules, where the angle between the renal tubules and pelvis is greater than 45 degrees, hard stones or ones in distal position in the renal tubules reduce ESWL efficiency. Extracorporeal lithotripsy as a monotherapy is a relatively more e&ective method in the paediatric population than in adults, because of the stones’ so+ness, their smaller sizes, the smaller volume of the patient’s tissues during shockwave transmission and the greater facility in spontaneous evacuation of the crushed stones. [17, 18, 19]. However, even a+er successful defragmentation of the stones, there is a risk that the crushed fragments will remain in the pelvic cavity or block the normal flow of urine creating a so-called stein strasse in the ureter (Figure 2). The most common complications seen a+er ESWL are: bleeding from the urinary tract, bruising, renal parenchymal haemorrhage, and renal colic. [16]. No late complications, such as worsening kidney function or hypertension, were observed. [20]. Vlajkovic et al. evaluated GFR kidneys before and a+er ESWL. The authors showed that GFR normalizes or grows approximately 3 months a+er surgery, which allows ESWL to be considered a safe procedure.

## Percutaneous nephrolithotomy (PCNL)

**Fig. 3:**
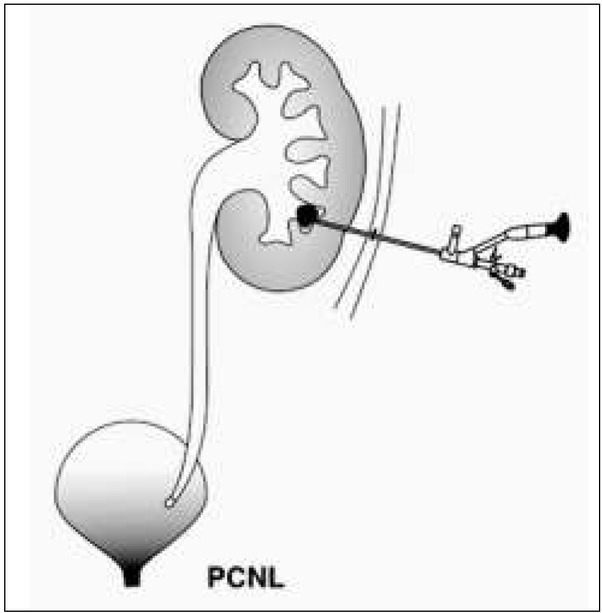
PCNL procedure diagram. Ryc. 3. Schemat procedury PCNL.

PCNL - percutaneous nephrolithotomy (Figure 3). This procedure is performed under general anesthesia and under antibiotic prophylaxis. Using radiological or ultrasound guidance, a renal calyx is perforated percutaneously, then a nephroscope is introduced and the stones are crushed. Pneumatic, ultrasonographic or laser (Ho:YAG) lithotripters may be used for stone defragmentation. According to recommendations of the European Society of Urology, PCNL is the method of choice in the treatment of:

- deposits ≥1.5 cm located in the upper pole of the kidney,
- deposits of ≥1.0 cm located in the lower pole of the kidney,
- hard stones, such as cystic or struvite ones
- stones in kidneys with anatomical defects that may hinder the flow of residual stones

Major complications associated with this procedure are: fever, urosepsis, and intense bleeding requiring blood transfusions. However, as the experience of authors from di&erent centers show, the risk of blood transfusion is very low [21, 22]. Dawaba et al. monitored renal function using dynamic renal scintigraphy. As demonstrated in 65 patients, renal function improved or remained unchanged (in all but one patient). The effciency of PCNL as a monotherapy varies from 87% to 98.5% [23, 24]. To increase the e&ectiveness of this method, in many health centers the so-called “sandwich therapy” was implemented, i.e. the ESWL procedure is additionally performed a+er the PCNL procedure. This way of treatment may attain an even 100% effciency [25]. Thanks to the miniaturization of equipment, many PCNL modications have been introducedcations have been introduced. Now there are mini-PCNL (miniperc), ultra-microPCNL (ultra-miniperc) instruments, as well as the most miniaturised microPCNL ones. During the procedure, 8/8.9 F nephroscopes are used in the 12 F shields. The instruments are used under the control of ultrasound, which makes it possible not only to avoid harmful radiation but also to accurately determine the anatomy of the renal pelvic system and locate the blood vessels, thus avoiding uncontrolled bleeding. PCNL is a technique requiring extensive surgeon experience but it may be a very e&ective method and is a good alternative to surgical treatment.

## Ureterolithotripsy (URSL)

**Fig. 4:**
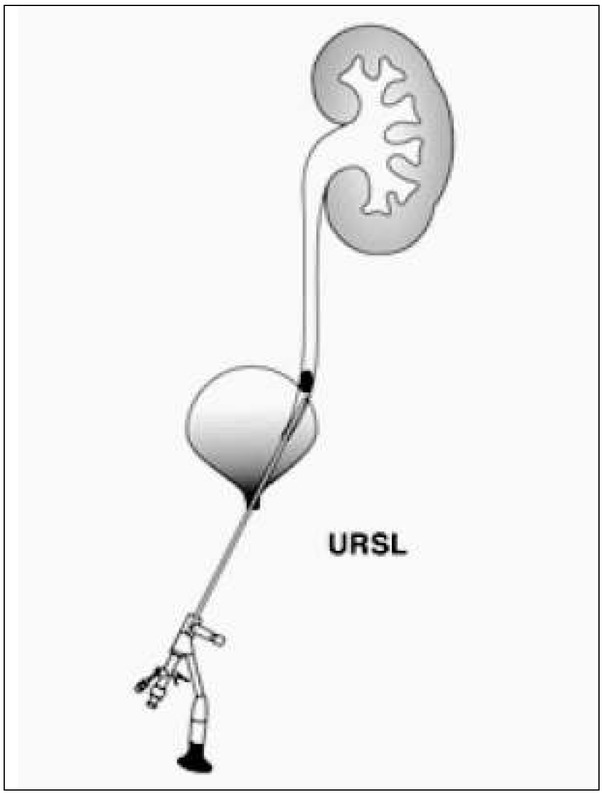
URSL procedure diagram. Ryc. 4. Schemat procedury URSL.

**Fig. 5:**
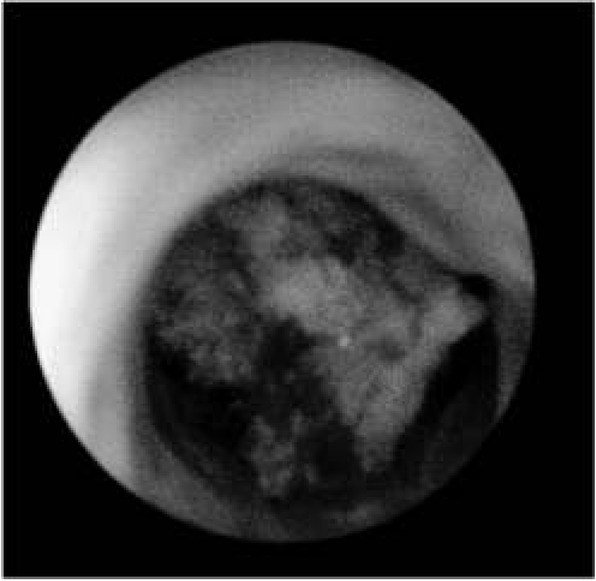
Stone in the ureter. Ryc. 5. Złóg w moczowodzie.

URSL (Figure 4) is a procedure that allows not only ureteroscopy up to the pyeloureteral junction (Figure 5) but thanks to the miniaturization of flexible ureteroscopes also the examination of the renal pelvis and secondary renal calyces with a possibility of crushing stones by the lithotripter -RIRS procedure. Pneumatic and laser lithotryptors are used for crushing. The method of disintegration of the stones is quite different. The ballistic lithotryptor allows the crushing of the deposit to very small fragments, and apart from this, the laser can also be used to perform the so-called dusting technique. The effciency of both lithotryptors is comparable. In the literature, Corcoran et al. received 88% effciency a+er a single procedure using Ho:YAG lithotripters in a group of 47 children with stones located in the upper part of the urinary system [26]. Cannon et al. presented a 76% effcacy of URSL in the treatment of stones with an average diameter of 12.2 cm in the lower renal tubes. Among complications related to URSL, there are: ischaemia, stenosis, ureteral perforation and vesicoureteral reflux. The risk of complications is inconsiderable, i.e. about 2-4% [27].

**Fig. 6:**
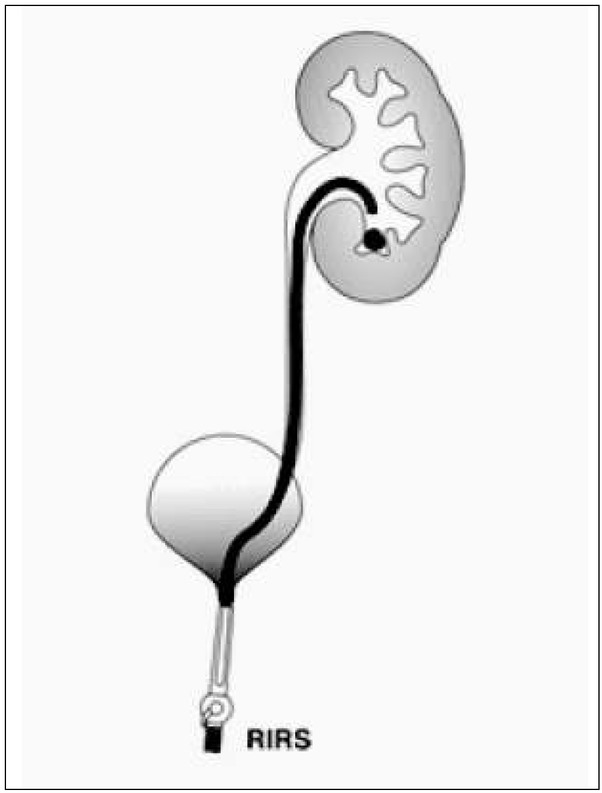
RIRS procedure diagram. Ryc. 6. Schemat procedury RIRS.

RIRS stands for retrograde intrarenal surgery (Figure 6). Due to the lack of suffcient miniaturization of flexible ureteroscopes, RIRS is still a very diffcult procedure to carry out in the youngest children. Jun Li et al. have performed RIRS in 55 infants using 8Fr/30 cm flexible ureteroscopes with very good results. The effectiveness of the method was 94.6%, and there were no serious complications associated with this technique [28]. The results are very promising, however they require more prospective studies. The development of minimally invasive techniques enabled the use of robotic techniques to perform endoscopic procedures more e&ectively. The Avicenna Roboflex robot is used to support the RIRS procedure to increase its effciency and safety. Nevertheless, there are few reports of this technique in children.

Pyelolithotomy as an open or laparoscopic method has a very narrow range of use and due to the significant development of minimally invasive techniques it is a very rarely used procedure. The main indication for open surgery is staghorn lithiasis in the renal pelvis and at least three groups of calyces. During a single procedure all the stones from the kidney can be removed without renal parenchyma damage. The number of pyelolithotomy has radically decreased. Despite this fact, open surgery plays a very important role in the treatment of urolithiasis in children. In those health centers worldwide which have appropriate medical equipment and a good team of experienced specialists, about 1-5.4% of patients with urolithiasis still require surgical treatment [29, 30]. The need for pielolitotomy in children is much higher (up to 17%) than in adults [31, 32]. This may be due to the di&erent characteristics of the stones, the size of the patient and the presence of anatomical anomalies requiring simultaneous surgical correction [33]. Open surgery is indicated in cases, when anatomical anomalies coexist with urolithiasis, i.e., sub-pelvic stenosis, or when the use of PCNL or ESWL is diffcult, or impossible.

**Fig. 7:**
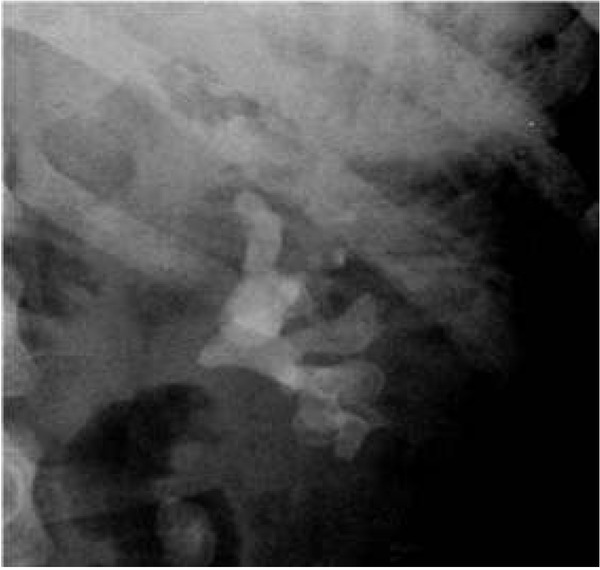
Staghorn lithiasis – plain X ray. Ryc. 7. Kamica odlewowa – RTG przeglądowe jamy brzusznej.

The authors have developed and described a new alternative method to classical open surgery. Combining pyelolithotomy with endoscopy to remove concrements clears the diseased kidney without causing parenchymal damage in one procedure. The method is safe in children, does not require blood transfusion, and helps maintain kidney function. This procedure is dedicated to complicated cases of staghorn urolithiasis [34] (Figure 7).

Due to its increasing incidence, urolithiasis is a serious interdisciplinary problem. It requires a thorough understanding of the causes, as well as e&ective and least invasive treatment. The ideal procedure should be e&ective, safe and allow the complete evacuation of the stones a+er the 1st procedure.
